# Reciprocity in Medical Licensure; A Plea for Interstate Indorsement

**Published:** 1897-07

**Authors:** William Warren Potter

**Affiliations:** Buffalo, N.Y.; Examiner in Obstetrics, New York State Medical Examining and Licensing Board


### BUFFALO MEDICAL JOURNAL.

Vol. XXXVI. JULY, 1897. No. 12.

### Original Communications.

### RECIPROCITY IN MEDICAL LICENSURE; A PLEA FOR INTERSTATE INDORSEMENT.

Presidential address before the National Confederation of State Medical Examining and Licensing Boards, at its seventh annual meeting, held at Philadelphia, Pa., May 31, 1897.

By WILLIAM WARREN POTTER, M. D., Buffalo, N. Y.

Examiner in Obstetrics, New York State Medical Examining and Licensing Board.

FOR the second time it becomes my duty to assume the functions of presiding officer over the deliberations of this confederation, and I beg, at the outset, to offer you my best thanks for this renewed mark of your favor.

### IN MEMORIAM—DR. PERRY H. MILLARD.

Since our meeting at Atlanta last year the confederation has suffered beyond measure in the death of one of its most able and useful members, Dr. Perry H. Millard, of St. Paul. Since the death of Dr. Rauch, I know of no one whose counsel has been so necessary to our welfare, and of none whose devotion to the interests of the organisation has been greater. It was my sad privilege to visit him at Johns Hopkins Hospital only a month before his end and then, though feeble in body, his clear and active mind was busy with the affairs of this confederation, and especially with the report of the committee on minimum standards, which he, as chairman, hoped to make at this meeting. But his final summons came on February 2, 1897, when this accomplished physician and useful citizen passed to his immortality. "His spirit was twenty, his years were fifty, but, alas! his body was eighty. Farewell."

### INTRODUCTION.

At our meeting last year it seemed proper to explain in considerable detail the objects of this organisation in order that there might be no misunderstanding as to its purpose. For some time previously the medical schools, or at least some of them, had looked with oblique eyes upon our movements, fearing, possibly, that we might be scheming to interfere with their prerogatives in the educational field. At first, too, some of them were inclined to criticise the provision made in most of the states that teachers in medical colleges shall not be eligible to appointment on examining boards. The wisdom of this proviso, however, must be apparent to every thinking man. No one disputes the fact that there must be absolute impartiality in the examinations, but how could this prevail if college teachers were made members? Obviously, one college would be jealous of another, and there would be no end of criticism and contention over the work as well as the representation on the boards; especially so in those states in which there are several schools.

It was further alleged in some quarters that none but teachers were competent to prepare questions or mark answers. The absurdity of this suggestion has been abundantly proven. As a matter of fact the test of the state examination is different from that applied to undergraduates. The state examiners must, by a few well-chosen and comprehensive questions in each branch, determine a man's general fitness to practise the science and art of medicine. They must so frame the few questions they are permitted to ask, and which there is but scant time to answer, so as best to develop that fact in a general way. On the other hand, teachers having been in close relationship with their pupils for three or four years need examine only on the subjects upon which they have given instruction; they often ask categorical questions, or those admitting of incomplete answers—a defect that is expected to be, and usually is, supplied in answer to the next question in further elaboration of the subject, which is easily understood to follow. This method is quite right and proper in a teacher's relation to the pupil or the candidate for the doctorate degree, but not admissible from our standpoint. Here each question must be clear, comprehensive and complete in itself, not susceptible of being answered by "yes" or "no." Ours is, or should be, essentially a post-graduate examination.

In order to test this matter, I have asked teachers, in a few instances, to submit groups of questions; but I have found them, speaking generally, not adapted to the state test, although quite satisfactory to ask at pass examinations or of candidates for the doctorate degree. A state examiner will soon acquire the skill of propounding questions in a fashion best adapted to his work, especially those that will best test the quality of instruction the candidate has received. Once this important and tactful knowledge is obtained, his services become of incalculable benefit to the state and he should be retained as long as he will consent to serve.

Another fear of the schools was that the high standards, directly and indirectly growing out of state control, would empty their benches, or that there would be such a diminution of pupils as to materially deplete their incomes. Quite the contrary has been the fact. Bellevue, at New York, graduated a larger class this year than in some years past and its lecture rooms are reported to have been full. The College of Physicians and Surgeons (medical department of Columbia University) has found it difficult to provide room for its overflowing classes. In the city of Philadelphia, which, since an early day, has been a center of medical education, the schools are full even to embarrassment. In Buffalo, the University Medical College never had so many matriculates nor so large a graduating class as this year; and so it is wherever high standards have been adopted. The explanation is not difficult: students want the best; it costs no more in the end and serves them with much greater satisfaction in their early struggles for recognition and professional favor, and enables them to maintain a higher position in later years.

It was made apparent at our meeting last year that the examiners compare favorably in education and personnel with the professors with whom we were assembled in joint session; and I hardly think it likely that we shall again hear the challenge, given probably more to cover a retreat than to mask an advance, "Who shall examine the examiners?" The answer, were one necessary, is not difficult to find; for the self-same authority that examines the examiners will, no doubt, find it an agreeable duty likewise to examine the professors.

No! there is no disagreement between the college faculties and the state examining boards, for it has been clearly demonstrated that they are friends. Their interests are common and their objects one. They must not be drawn into antagonism by the indiscreet utterances of a few misguided though well-meaning persons. Each is working in separate yet contiguous fields, and all are striving for the same purpose—namely, to obtain the best possible equipment for every man and woman sent out over this broad land to practise the profession of medicine.

There is, then, less occasion for extended general remark now than heretofore, as I have already intimated ; hence I shall address myself to the discussion of but a single topic, one, however, of paramount importance.

### INTERSTATE EXCHANGE OF LICENSES—THE PROBLEM.

There are at this moment two questions pertaining to state control of medical practice that seem to tower above all others in the minds of many physicians, which demand our most careful attention and challenge our deliberate judgment. The two are, however, so inseparable that in reality they may be considered as integral parts of one subject and discussed together. I refer to (*a*) minimum standards of requirements to enter upon the study of, acquire a diploma in, and obtain a state license for the practice of medicine ; and (*b*) to the interstate indorsement or recognition of licenses so that, under prescribed rules, a licensee of one state may be permitted to practise in any other state in which he may seek a temporary or permanent residence.

It goes without saying that an exchange of these official courtesies between the states "is a consummation most devoutly to be wished" by every friend of state control in medicine. It is one of the principal objects that this confederation is laboring to accomplish. It is at the same time one of the most difficult problems thrust upon us for solution. No one can deny the fact that it is pleasant to contemplate a time—I trust in the near future—when we may have a national registration bureau where every legally qualified reputable physician may be recorded, and when all physicians whose names appear on this register and whose licenses are properly indorsed by the registrar, may pass from one state to another in the practice of their profession and in the enjoyment of all the privileges thereto appertaining.

But how is this to be established with celerity and with justice to all concerned ? The idealogues who were chiefly interested in the agitation of the question of reciprocity of licensure, assert that unless an interstate exchange is arranged, and that speedily, the whole system of state control in medicine will go to the wall. Whether this is given out in the nature of a threat or a prediction I know not, but there are indications that lead me to suspect the former. These men I believe are, for the most part, specialists who spend the vacation months at summer resorts, watering-places, or on comfortable farms that their plethoric purses have secured in states more or less remote from their homes. They expect to make a snug sum from consultations and office patients during the vacation season, but do not relish the idea of being compelled to "undergo the nuisance of examinations," as they characterise it, in the states they have chosen for their holiday practice.

They are frequently men of influence who seek to prejudice their medical friends against the system, by pointing out what they are pleased to term the injustice of applying the state laws in their cases, and then proceed to air their grievances in the columns of the medical journals as a court of last resort. While I have no doubt that the denial of reciprocity now and then works hardship to deserving men who perforce must change their residences, but can ill afford to spare the time and money to take a new examination, I yet fail to see wherein the class of men first above referred to and who prate the loudest about it, are deserving of special sympathy. They can well afford to submit to an examination in the states were they pass a profitable and easy summer ; moreover, I am unable to discover any injustice in compelling them to comply with existing laws. Shall a state require of its own citizens a compliance with its practice laws and at the same time grant to the well-to-do summer specialist exemption from their operation ? As the state laws, for the most part, forbid discrimination against the inhabitants of each, there is both a moral and a legal bar to such exemptions. Let us, however, calmly examine the question with a view to arrive, if possible, at an intelligent and just solution of the problem.

### EQUALITY OF STANDARDS A BASIS FOR RECIPROCITY—THE OBSTACLES.

The only equitable basis upon which reciprocity can be established that appears both feasible and practicable, is that of equality of standards for admission to the study and practice of medicine. This implies an equalisation of the preliminary requirements of medical students and a uniformity of applying the tests ; a uniform period of collegiate training, including uniformity of methods of teaching ; and, finally, an absolute similarity in the methods of conducting state examinations and granting licenses. A minimum standard of preliminary qualifications is important to agree upon. This ought to be done in a uniform manner by all the states for the sake of the good name of our profession, even if there were no other cogent reasons demanding it. American medicine should not be disgraced any longer by illiterate physicians ; hence, illiterate students ought not to be admitted to our medical schools. If we cannot agree upon any other question we ought to demand this much as a united profession.

What, then, shall be the minimum limit of education below which a student of medicine shall not be accepted ? No more important question awaits answer, but it is one on which, I am sorry to say, there is much diversity of opinion. If a man's mind has not already been disciplined to the extent of acquiring a good English education before he takes up the study of medicine, it presents a sorry foundation upon which to engraft a knowledge of such a multiform science and art. It is far more important, in my view, to agree upon a reasonably conservative standard of minimum requirements than it is to insist upon extending the terms of medical teaching to four years ; since it is better to teach three years of medicine to a well-disciplined mind than to demand four years' medical training of an illiterate student. It is to be hoped that this cardinal principle can be definitely settled to the satisfaction of all the states at an early day.

But let us pass to the next topic—namely, uniform periods of collegiate training. Here, fortunately, there is less diversity of opinion, thanks to the good work of the Association of American Medical Colleges. Under the stimulating influence of the American Academy of Medicine, well seconded as I believe by this confederation, we find a public sentiment fast settling to the conclusion that four years is quite short time enough in which to acquire the proper training and knowledge to fit one for the doctorate degree. It must be remembered that it is required nowadays of the undergraduate to pursue courses in anatomic, physiologic, chemic, biologic, pathologic and bacteriologic laboratories, and to undergo training in medical, surgical and obstetric clinics ; add to this the necessary didactic and recitative courses besides training in special techniques,—microscopy, ophthalmoscopy, laryngoscopy, and the like,—with all these, besides much other information that must be acquired, and who shall say that four years is too long a time to devote to college work ? It would be interesting in this relation to trace the progress that has been made in the last quarter of a century in the various departments of medicine, but they are as familiar to you as to me. You know how the complexity of the present age with its rapid transit and instantaneous methods of communication has correspondingly increased assaults by the enemies of health that, too, are multiplying with such marvelous rapidity. To deal successfully with this complexity requires a mind more deeply and broadly cultivated; preparations for receiving and utilising medical instruction must be increased and lengthened; as our civilisation develops our immunity from disease lessens, hence our vigilance and efficiency must be greater, our period of preparation longer, and our training increased in length, depth and breadth. It is, therefore, with pleasure that we observe and heartily approve the good work of the association of American medical colleges that is arranging the curricula in such a manner as best will be adapted to the environment of the present day. In this relation it would also appear essential that methods of teaching should be reduced to a degree of uniformity that heretofore has not been attained. With a universal establishment of four years' courses in American medical colleges, it will become appropriate for them to grade their curricula to as near a common level as may be consistent with surrounding conditions. Methods of teaching in all the laboratory departments could easily be conducted on a scale of similarity that would make them practically uniform in all the colleges. Then it would matter little where the student received his doctorate degree, as all colleges would be on or near at par.

The third condition to be standardised is one that immediately concerns this confederation and it becomes us to deal with it in a most thoroughgoing manner. The first step toward equalising methods of applying the separate examination for license in the several states, it seems to me, is to bring the examiners together on the subject. If they can be made to agree upon questions concerning their all-important function, then we have gone a long way toward establishing uniform methods. If, however, they differ in opinion as to the application of the principle in the several states, the prospect for speedy adjustment presents a more discouraging view. The first point that offers itself is so plain that I am sure no one will attempt to dispute it. The foundation principle of a separate examination for license to practise is that it shall be applied to all with absolute impartiality; there must be no exceptions to the rule, no exemptions.[1] While it is true that a law on

1. If some who demand exemption would spend as much time in preparing for the state examination as they do in trying to avoid it, they would command more respect, and be better off.

this subject cannot be made retroactive and that all legal practitioners at the date of its passage must continue to be so recognised, yet all others must be examined ; and so the work must continue from year to year. Another inflexible rule that ought to prevail is that a diploma from a registered school should be demanded as a passport to the state examination. The logical conclusion is that a state examination is supplemental and consecutive to that of the schools, and that it should be refused in all cases where applicants are not holders of diplomas legally obtained from registered and recognised colleges. If this rule were scrupulously enforced it would deprive medical college faculties of grounds for any further opposition to state control. If, on the other hand, any of the state examining boards are permitted to examine undergraduates for license, it enables them to set up standards of their own—a function antagonistic to the underlying principle of state control and, I fear, subversive to the best interests of reform in medical education. When the duties of medical examiners are reduced to the mere question of determining the qualifications of such individuals as legally constituted schools shall turn over to them with M. D. degrees, they will have served the whole purpose for which they have been created. It is understood, of course, that there must be a uniform system of recognising and registering the medical schools in the several states.

With the foregoing principle once settled the minor details of standardising state medical examinations for license could be more easily arranged. A uniform system of propounding and marking questions becomes desirable. For instance, if it could be agreed that ten questions in each department or topic should be asked and that each answer should have a possible value of ten points; and, further, that the total possibilities of the examination should be fixed at 100 points, maximum, and 75 points, minimum, in each topic, we could easily approach uniformity in this part of our work. The only remaining important question would be as to the valuation of the answers. This necessarily will always vary a little in degree, since the personal equation of the examiner enters somewhat largely into its outcome. It is a well-known fact that some examiners are inclined to a high, others to a medium, and still others to a low valuation ; but with experience and an interchange of opinion on the subject this factor would soon be reduced to an exiguity that would render it comparatively unimportant.

### LEGISLATIVE ENACTMENTS—THE SOLUTION.

These, then, are the essential steps towards reciprocity; these are the obstacles to be overcome before it can be accomplished. The remedies lie in legislative enactments and these, speaking generally, are of slow development. Nevertheless, with a healthy public opinion once aroused on the subject, legislative bodies will soon take heed and adopt adequate measures to overcome defects. The newer states are likely to fall into the front line sooner than the older ones that already have imperfect laws. Public opinion moves faster, and I had almost said in a sounder fashion, as we travel toward the Occident. This, in some respects, is no doubt true. Montana, for instance, stands at the forefront in all of its requirements for state license. Minnesota, too, has been a pioneer in the movement for advanced standards and has one of the best laws. Moreover, we may expect those states east or west, north or south, that as yet have no laws relating to medical practice, taking heed of the necessities arising under present conditions and profiting by the experience of those states that have preceded them in relation to state licensure, to enact laws that will meet all the conditions of the present day and which will satisfy the most idealogistic views.

At present, twenty-seven states demand separate examination for license to practise medicine. In fifteen of these a legally obtained and possessed diploma is the first condition imposed; without it a candidate cannot be admitted to the examination. It only remains for this latter group, by statutory enactments, to bring their preliminary requirements to a common level and then for their several examining boards to agree upon uniformity of methods, when, lo! the question of reciprocity is solved. A license granted by one of these states will then be valid in all others of this class, upon proving identity, character and the payment of whatever fee may be imposed. With reciprocity once accomplished between these states, the others, one by one, will soon afterward establish themselves on the reciprocity basis in self-defense, if for no other or better reason.

Those who most loudly and persistently demand interstate indorsement aim their criticisms at the examining boards, holding them responsible for all their woes, whereas, as a matter of fact, the examiners have nothing whatever to do with the question. They are simply agents of the states to administer the laws as they find them and cannot change the practice in regard to reciprocal interchange of registration. The statutes in every instance with which I am familiar merely permit the acceptance and registration of licenses issued by other states, where the standards are at least equal in all respects to those of the state issuing the license. This means, if it has any significance whatever, that in all preliminary requirements, in collegiate training and in state examinations, one and all, there must be an equality and a uniformity of standards before licenses can be accepted for registration in a reciprocal manner.

The precise language of the New York statute on this subject is as follows :

Applicants examined and licensed by other state examining boards registered by the regents as maintaining standards not lower than those provided by this article . . . . may, without further examination, on payment of $10 to the regents, and on submitting such evidence as they may require, receive from them an indorsement of their licenses or diplomas conferring all rights and privileges of a regent's license issued after examination.

In an amendment to the practice law passed March 21, 1896, this principle was reaffirmed in the following terms :

New York medical schools and New York medical students shall not be discriminated against by the registration of any medical school out of the state, whose minimum graduation standard is less than that fixed by statute for New York medical schools.

As many other states have enacted statutes fashioned after that of New York, containing this particular proviso, and as still other states hold to the same provision in effect, it is easy to understand how powerless the examiners are in the premises. These demands of the restless and migratory doctors must be taken to the state legislative halls and there made known, if relief is expected. Meanwhile, the members of this confederation may assist in bringing the problem to more speedy solution by acquainting their legislatures with the difficulties to be overcome and by urgently recommending the adoption of such amendments to existing laws as will meet and remove the present defects.

My object in discussing this subject in detail is to place the examiners right before the country in regard to it and to divert further criticism against the delay of reciprocity into the proper channel.

### PROGRESS DURING THE YEAR.

During the past year attempts have been made in nine states and territories to establish medical examining boards, in four of which efforts have been successful. Those that have joined the list with practice laws are: District of Columbia, Idaho, Indiana and New Hampshire. The laws of New Hampshire, District of Columbia and Idaho make a diploma from a registered school a requisite to enter the examination. In Indiana the law is modeled somewhat after that of Ohio, and is a substantial beginning in a state that has sadly needed protection from charlatans and medical pretenders. So, in the academic year just now about to close, there has been substantial gain in the direction of state control, even if some important states have fallen "just outside the breastworks." These no doubt will re-form their lines and come to the attack another year with full promise of success. If four states and territories are added to the list each year, in a very short time we shall be able to rejoice in a professional rehabilitation that will compel the admission of the American licentiate to the best medical standing wherever civilisation exists.

It seems important in view of the present condition of state control that the committee on minimum standards, appointed last year, shall be continued as a standing committee of this confederation, with enlarged powers that shall include also the question of reciprocity. Permit me to invite your attention to a brochure, entitled, "State requirements for the practice of medicine; a guide to the qualifications and methods of procedure to enter upon the practice of medicine in the United States," compiled and published by Dr. Charles McIntire, of Easton, Pa. It constitutes the Bulletin of the American Academy of Medicine for February, 1897, and is by far the most complete publication on the subject yet issued. If secretaries of boards had paid more attention to requests for information the book would have been absolutely accurate up to the date of its issue; as it is, it is surprising how much of value Dr. McIntire has been able to furnish. Every state examiner and especially every secretary should be supplied with this guide and it should be placed on file in all public libraries.

QUESTIONS OF PUBLIC HEALTH ARE INVOLVED—CONCLUSION.

To return for a moment and finally to the special subject-matter of this paper: I venture to promise, that in any event and under all circumstances the honest and earnest friends of reciprocity may be sure of the active and zealous coöperation of this confederation in all reasonable efforts to establish it. In most of the states where state license is established the struggle to obtain even imperfect laws has been prolonged and the contest has been exasperating as well as fatiguing. Every possible influence has been brought to bear on legislatures to prevent even a conservative recognition of the principle. Nor is this all : after laws have been passed making a good start in the direction of this reform, the state medical societies, through whose influence, speaking generally, the measures have been adopted, have been kept constantly on the defensive at legislative hearings and otherwise, to prevent amendments to existing statutes that would weaken or tend to destroy their usefulness.

These proposed amendments generally have their origin in spite, malice, caprice, or vengeance, and should always be regarded with suspicion by legislative bodies. It will be a happy day for the friends of state license when legislatures can be persuaded to turn a deaf ear to all amendments that are proposed outside of state medical societies or state boards of medical examiners. They know the defects in statutes and can be trusted to formulate the remedies. This would save much time to members of legislative bodies and give security of feeling to the medical profession. If legislators, too, could be made to appreciate the fact that public health interests are involved in the question of state license; that every attempt to weaken the principle is a blow at public sanitation ; and that higher standards of medical education mean better health for the people, then, perhaps, it would be easier to obtain and maintain the necessary laws to protect the commonwealth against that kind of ignorance, superstition, or super-refinement that always lurks in the environment of quackery.

Traveling and advertising quacks, christian science and faith healers, clairvoyant and spiritualistic mountebanks—these and every other kind of pretender that panders to the ignorance and superstition of the people, leeching their money and sapping their vital force, ought to be no longer tolerated by enlightened communities ; and if legislatures would lend a willing hand these villainous enemies of public and individual health and prosperity might soon be disposed of in a fashion becoming an intelligent and progressive age.

284 Franklin Street.

